# Exhibitionist Eating: Who Wins Eating Competitions?

**DOI:** 10.3389/fnut.2016.00051

**Published:** 2016-11-24

**Authors:** Brian Wansink, Kevin M. Kniffin

**Affiliations:** ^1^Charles S. Dyson School of Applied Economics and Management, Cornell University, Ithaca, NY, USA

**Keywords:** competitive eating, gender, impression management, evolutionary psychology

## Abstract

**Objective:**

How and why does competition and spectator involvement influence eating behaviors? The primary objective of this article is to explore the nature of competitive eating with the goal of identifying implications for other social situations.

**Design:**

Study 1 investigated how many chicken wings were eaten by men and women in a 30-min eating competition when cheering spectators either were or were not present (compared to a control condition). The second study sought to explain Study 1’s findings through a survey of 93 students who rated male or female competitive eaters (in randomized order) based on intelligence, attractiveness, health, strength, and how romantic they expected the eaters to be.

**Results:**

Exploratory findings show competitive eaters ate approximately four times as many chicken wings as a similar control group, and the presence of a cheering audience further increased wing consumption for males (but decreased consumption for females). Study 2 suggests part of the over-performance of males may be related to a shared positive perception that competitive male eaters are strong and virile.

**Conclusion:**

Even in relatively low-stakes environments, competitive visibility may dramatically increase how much males eat. These preliminary results help illuminate recent discoveries that males overeat in various social situations where there are opportunities for men to “show off.” This may have relevance for dining behavior – especially among younger males – at parties, banquets, group dinners, and similar social situations.

## Introduction

While the factors that motivate people to enter eating competitions or food challenge are fairly clear – fun, variety, acknowledgment, ego enhancement, prizes, and so on – it is less clear what factors influence a person’s performance (1–4). One factor could be the competitive desire to win (5) and to receive the related acknowledgment (6). The second factor – building upon research that shows that people eat more with companions (7) – might be the reinforcement or attention that comes from the presence of a crowd or spectators and the excitement and energy they can provide (8, 9). How do these two factors – competition and spectator support – alone and jointly impact the quantity of food eaten in competitions?

Recent findings have shown that young males have been particularly prone to overeat in some social situations where there are either (a) models to suggest that overeating is normal or (b) prompts to invite men to “show off” and emphasize their masculinity by eating meat (10) or even dogfood (11). For instance, when dining out, young men have been shown to eat more desserts and drink more alcohol when in the presence of an obese waiter (12) or in the presence of an obese colleague (13). With a different kind of prompt, men eating at all-you-can-eat pizza buffets with a female dining companion ended up eating twice as much pizza and salad as those in the arguably less “show off” context of eating with another male (14).

In contrast with research documenting overconsumption by men, studies of disordered eating among women tend to focus more on underconsumption. For example, women have shown a tendency to eat less than they would normally eat in the presence of a man (15). This has been proposed to be due to impression management, but it could also suggest a general tendency to eat less when under scrutiny (16). Recent research has shown that men tend to eat more in the company of women perhaps to signal their masculinity (14), but it is interesting in relation to our interests that the prior work was limited to relatively private eating in discreet situations.

Far from being either private or discreet, competitive eating is one of the most extreme illustrations of conspicuous consumption. In fact, it often plays a comical role in American culture; and, as described closely by Nerz (1), eating competitions are growing in frequency, size, and variety of food-types over the past two decades. The emergence of competitive eating raises important questions of what motivates exaggerated levels of consumption. Knowing this could illuminate how similar motivations might influence certain people in more common social situations – such as parties, banquets, group dinners, and other intense social situations (17, 18). By exploring behavior in an ostensibly competitive-eating environment (Study 1) and measuring perceptions of competitive eaters (Study 2), we hope to better understand non-functional competitive eating.

## Materials and Methods

### Study 1: How Eating Competitions Influence Consumption?

University students of similar height and weight were recruited to be involved in an IRB-approved study involving an eating competition for which they would “publically receive a token medal that is symbolic but has no cash value.” They provided written informed consent and were awarded extra course credit for their participation.

More specifically, volunteers who said they had an interest in competitive eating were recruited from three upper-division courses in biology, consumer behavior, and public affairs (approximately 18% of the total population of students). After completing a brief screening questionnaire, 24 people were invited to be involved in the study based on them having eaten chicken wings at least three times in the past 3 months, and based on them having s similar body mass index (BMI 22.5–27.5) and age (21–22). They were instructed to eat a normal breakfast on the day of the study (2 days after being invited). On the day of the study, two individuals did not appear on time, and two others reported they had not eaten breakfast and were dismissed. The remaining 20 volunteers were given lunch in exchange for their participation.

For the three conditions that we examined through Study 1, participants were randomly assigned to be either (i) competitors in a chicken-wing eating contest, (ii) competitors-with-cheering-spectators in a chicken-wing eating contest, or (iii) part of a larger non-competing control group invited to eat chicken wings.

Two males and two females were randomly assigned to the (i) competition-only condition that did not have any spectators. They were told that they were competing with another team and that they would win a modest commemoration medal if their team collectively ate more chicken wings than the other team in 30 min. The two males and two females randomly assigned to the (ii) competition-with-spectator condition were given identical instructions, but their 30-min eating session took place in the presence of 12 cheering and applauding spectators comprised by colleagues and research staff.

Participants in both competitive-eating conditions – (i) and (ii) – were supplied with an unlimited supply of chicken wings along with French fries, coleslaw, and soft drinks. Participants were informed that the competition was based solely on the number of chicken wings they ate; the other foods were provided for variety. The 12 participants in the (iii) control condition were randomly assigned to a seat and were provided with the identical food in addition to being invited to eat as much as they wanted within 30 min.

For all the three groups, consumption was individually tracked based upon the total number of residual chicken bones on their plates. French fries, coleslaw, and soft drink consumption were tracked in aggregate terms of how much was served and how much was consumed by each group in total.

While most participants completed the full study, one person was eliminated from the control group because upon being served lunch, they announced they had an emergency phone call and left the room without eating. Consequently, 11 people – 4 men and 7 women – finished their lunch in the (iii) control condition.

At the end of the 30 min, participants in both treatment groups were asked to provide open-ended verbal feedback to researchers in a debriefing. Following the oral descriptions that participants provided concerning their experience in the study, participants were publicly awarded inexpensive plastic gold or silver medals to commemorate their performance.

## Results and Discussion

*F*-tests were used to compare how many chicken wings that men and women in the treatment groups and the control group consumed. For our main analysis, we conducted a 2 (gender) × 3 (condition) ANOVA. Main effects are significant for both gender [*F*(1, 14) = 42.9, *p* < 0.01] and condition [*F*(2, 14) = 91.7, *p* < 0.01]. More notably, the interaction of the two variables is significant [*F*(2, 14) = 14.0, *p* < 0.01]. In order to consider the possibility that wing consumption was non-normally distributed, we calculated measures of skewness (1.11, SE = 0.51) and kurtosis (0.08, SE = 0.99) – both of which are within reasonable ranges for not violating assumptions associated with the analyses that we conducted (19).

To complement the 2 × 3 ANOVA to better understand the relevant patterns, we conducted a one-way ANOVA with the factor gender that shows that – across the full sample – male participants ate more than female participants (*M*_men_ = 15.88, SD = 11.42; *M*_women_ = 7.36, SD = 5.97); [*F*(1, 18) = 20.74, *p* < 0.001; Cohen’s *d* = 0.95].

Similarly, when we combined data from conditions (i) and (ii) for a one-way ANOVA compared with the control (iii), we find that both men and women in the eating competition conditions – (i) and (ii) – tended to eat significantly more than those in the control [*M*_i + ii_ = 20.6 (SD = 7.53) vs. *M*_iii_ = 3.9 (SD = 1.44)] wings; [*F*(1, 18) = 114.97, *p* < 0.001; Cohen’s *d* = 3.08]. More specifically, a one-way ANOVA with the factor gender that is based on the sample of those in conditions (i) and (ii) shows that men in a competition-condition ate significantly more than women in a competition-condition group [*M*_men_ = 26.8 (SD = 4.71) vs. *M*_women_ = 14.5 (SD = 4.03)] wings; [*F*(1, 7) = 11.56, *p* = 0.003; Cohen’s *d* = 3.81].

**Figure 1:**
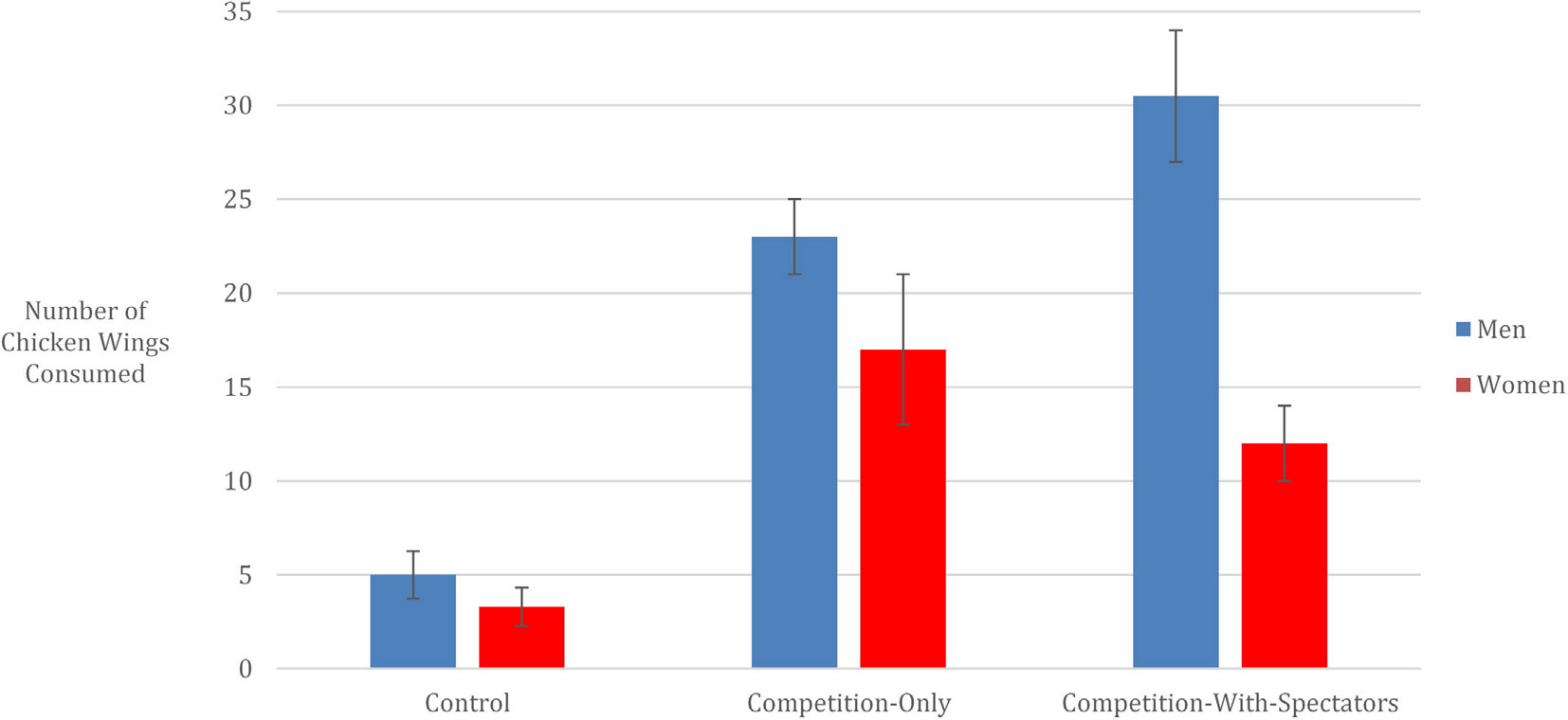
**The presence of a crowd encourages competitive eating (and overeating) among men more than women**.

Figure 1 helps to illustrate the patterns examined through our *F*-tests and shows how the presence of cheering spectators led male participants to eat an average of 30.5 (SD = 3.5) wings in condition (ii) vs. 23 (SD = 2) wings in the (i) competition-only condition (Cohen’s *d* = 3.75). Yet, this same cheering crowd led to an opposite reaction by female participants – women in the (i) competition-only condition ate 17 (SD = 4) wings, whereas women in the (ii) competition-with-spectator condition ate only 12 (SD = 2) (Cohen’s *d* = 1.58).

When participants were debriefed, the women in both the (i) competition and (ii) competition-with-spectator conditions mentioned feeling “self-conscious” and “a little bit embarrassed.” Men in the same condition did not use such descriptors of their experience, but instead used words and phrases with positive connotations like “challenging,” “cool,” and “exhilarating,” or “really a rush.”

Although these findings are exploratory, the influence of being in a competitive-eating situation was surprisingly strong. Yet in viewing the results in Figure 1, it raises the issue as to whether the additional impact of being publically cheered may be motivating for males but demotivating for females. To examine this, Study 2 will elicit the general perceptions of people in a similar demographic profile of those in this study.

## Materials and Methods

### Study 2: Male Competitive Eaters Are Perceived as More Reproductively Fit

To complement Study 1, Study 2 measured perceptions toward male and female competitive eaters regarding a variety of dimensions, including attractiveness, strength, and expected reproductive fitness. If people view men who are competitive eaters as more attractive on certain traits compared to those who are not, it would be consistent with the notion that increased encouragement and visibility help explain overconsumption by men. In contrast, if people view women who are competitive eaters as less attractive (20), it would indicate that encouragement and visibility might not be as motivating for them.

To explore this, 93 undergraduate students (34 women) from the same university and the same demographic pool as those in the Study 1 were recruited to participate in the IRB-approved study in exchange for partial course credit. Prior to asking for any assessments, we noted to participants that “Competitive eating has become relatively popular over the past 10 years, starting most famously with the Hot Dog Eating contest that is held each year on the 4th of July and which is broadcast on ESPN (a sports-based cable network).” We also highlighted that men and women have both been very successful in these types of contests.

Participants were then asked to rate – on a 9-point Likert scale – how they would rate a male or female competitive eater with respect to whether they were intelligent, attractive, romantic, healthy, and strong (1 = strongly disagree; 9 = strongly agree). Participants were asked to rate male and female competitive eaters in a randomized counter-balanced order. Following this, participants were asked to estimate the expected reproductive fitness of a male or female competitive eater. Specifically, we asked participants “Assuming that the average adult in the United States today is likely to be the parent of 2 children by the age of 50 years old, how many children would you expect a Male (*Female*) competitive eater to have by age 50?”

The question regarding reproductive fitness was generated from the perspective of evolutionary psychology whereby the possibility is considered that contemporary preferences for different types of consumption reflect patterns that evolved over the course of human evolution [e.g., Ref. (21–26)]. More specifically, Study 2 permits us to consider the “sexual selection” concepts of (a) intra-sexual selection, which predicts competition among members of the same sex and (b) mate choice [e.g., Ref. (27)]. If we were to find that women estimate male competitive eaters to have high reproductive fitness, then that would fit with a mate choice perspective that women tend to prefer men who are capable of substantial overconsumption. If, on the other hand, we were to only find that men estimate male competitive eaters to have high reproductive fitness, that would fit with an intra-sexual selection perspective that men are effectively competing with each other through eating competitions even if there is no benefit to be gained through the eyes of women’s preferences.

## Results and Discussion

For our initial analyses, we conducted *t*-tests to see if men and women viewed the competitive eaters differently. At that level of comparison, there were two differences. Men rated female competitive eaters as significantly less romantic [*M* = 2.24 (SD = 1.48) vs. 2.88 (1.56); *t* = −1.9*9, p* = 0.05; Cohen’s *d* = 0.42] and less attractive [2.47 (SD = 1.47) vs. 3.18 (1.57); *t* = −2.17, *p* = 0.033; Cohen’s *d* = 0.47] than female raters estimated the female competitive eaters. Given the 9-point scale, it is worth highlighting both men and women rated female competitive eaters relatively low on the two dimensions.

For finer-grain analyses, ratings from men and women were analyzed separately to test for differences in the way that members of each sex rated the male and female competitive eaters. Notably, *t*-tests showed that males positively perceived competitive-eating males to be both stronger [*M* = 5.15 (SD = 1.99) vs. 4.66 (2.20); *t* = 2.20, *p* = 0.032; Cohen’s *d* = 0.23] and having more offspring [*M* = 3.07 (SD = 0.83) vs. 2.58 (1.18); *t* = 4.11, *p* < 0.001; Cohen’s *d* = 0.48] compared to female competitive eaters. In contrast, women did not provide significantly different estimates of offspring count or general strength when comparing male and female competitive eaters. Likewise, the other variables that we measured did not vary significantly for this set of analyses.

These findings are consistent with the “show off” findings that Kniffin et al. (14) report whereby men – unlike women – appear to view conspicuous consumption of food to be a signal of fitness and success. As previewed above, the findings can be understood to recognize the role of intra-sexual selection rather than female mate choice since women do not appear to be favorably impressed by the feats of overeating.

## General Discussion

Although eating competitions and restaurant challenges around the world initially appear only as eating exhibitionism, they may hold insight into general everyday behaviors. For example, in Study 1, even a seemingly inconsequential eating competition – winning a $1.29 medal – led eaters to consume approximately four times as many chicken wings as a control group.

In combination, these two exploratory studies provide unexpected initial insights into how competition and spectators impact eating behaviors, but their impact may not be equal for all individuals. Whereas the presence of spectators in a competitive setting may lead some people to eat more, it may also cause self-conscious people to eat less. Indeed, this effect does appear to be mediated by gender, but the sample size of Study 1 was too small to be conclusive. A larger sample size is needed to determine if this difference was in fact due to gender or rather to individual personality traits of the participants, such as self-consciousness. Moreover, it may be that spectators cued to mind the competitiveness of athleticism, which has been shown to lead to exaggerated eating in the case of males watching exercise ads (28), thinking about exercising (29), or in reframing an activity as exercise or as a competition rather than as fun or as an adventure (30).

Study 2’s application of concepts from evolutionary psychology help to illustrate potential ultimate-level reasons for overconsumption by men in front of others. As compared with proximate-level reasons – environmental factors such as lighting, noise, or music, evolutionary psychology presumes that ultimate-level explanations such as intra-sexual selection do not require that people are conscious of the motivations that are hypothesized [e.g., Ref. (31–33)]. In the same way that a professional billiards player does not need to know complicated physics in order to excel at the game, our consideration of evolutionary concepts in relation to eating behaviors does not presume that men or women consciously or regularly think about reproductive fitness as a goal. Our interests in this regard also fit with more general analyses that recognize the potential signals that might be indicated by individuals’ food choices [e.g., Ref. (34)].

## Limitations and Future Research

As an exploratory examination of eating competitions, these two studies provide new insights that can now be further investigated with more subjects and greater confidence. Importantly, the powerful effect sizes involved in these investigations will probably require smaller sample sizes than are traditionally required to detect more subtle effects. Future research related to Study 1 should also, though, seek more diverse participant samples that include, for example, individuals who do not have a preexisting interest in competitive eating.

As with most eating contests and competitions, it is notable that Study 1’s primary interest was the total number of items (chicken wings) eaten and not with the total number of calories. While we assumed that this is roughly comparable, future research could investigate before and after weights. Similarly, we were not interested in the consumption of companion foods (French fries and coleslaw) and beverages; however, future research could examine non-focal foods and drinks more closely. Additionally, because prior studies showed that biting food off of a bone (vs. eating with a knife and fork) caused more aggressive behavior and uninhibited action (35), people were given chicken wings instead of pizza or other foods that would have generating less biting and more chewing.

With respect to Study 2, we did not control for participants’ familiarity – and appreciation – for competitive-eating contests though our prompt was designed to include basic background information to provide a common stimulus for the ratings we requested. It is imaginable that fans who attend and follow competitive-eating contests view the “athletes” differently than non-fans, and it would be valuable for future research to establish deeper understanding in relation to the eating contests.

Potential alternative explanations for the findings that we report in Study 2 include the view that “competitiveness” is what raters were implicitly considering. In that view, prior work has shown sex differences in competitiveness with men scoring as more competitive across a wide array of domains [e.g., Ref. (36, 37)]. Future research related to Study 2 will need to disentangle this potential confound more closely.

## Conclusion

Even in relatively low-stakes environments – competing for a $1.29 “Gold” or “Silver” medal – visibility and attention dramatically increase how much men will eat, but interestingly correlate with lower consumption by women. For men, this suggests a warning to many who would otherwise tend to overconsume in highly visible social situations, such as parties, barbeques, tailgates, receptions, and on dates.

Given the well-documented negative relationships between regular overeating and health, our findings suggest that men tend to “lose” eating competitions to the degree that men appear to be most likely to overeat in the company of an audience. While Study 2 considered the possibility that women might prefer men who can eat large quantities of food, our findings instead suggest that male overconsumption in front of spectators is a non-adaptive mismatch that does not directly benefit competitive eaters.

On a larger level, this article raises a broader question that is more cultural than caloric: why are eating competitions and food challenges so much more popular in the United States than elsewhere? Whereas Americans tend to embrace individual achievement and recognition by others, idiomatic expressions such as “The tall poppy gets its head cut off” are not part of the national mindset. Eating competitions and food challenges may be a venue by which individuals can distinguish themselves from others while simultaneously providing entertainment and amusement to an audience (38). In the end, this exhibitionist eating may say less about American eating habits than American ego habits.

## Consent to Publish

All the procedures performed in studies involving human participants were in accordance with the ethical standards of the institutional and/or national research committee and with the 1964 Helsinki declaration and its later amendments or comparable ethical standards.

## Author Contributions

BW designed and conducted the research, provided essential materials, analyzed data, wrote the paper, and had primary responsibility for final content. KK designed and conducted the research, analyzed data, and wrote the paper.

## Conflict of Interest Statement

The authors declare that the research was conducted in the absence of any commercial or financial relationships that could be construed as a potential conflict of interest.
